# Clinical outcome of tyrosine kinase inhibitors alone or combined with radiotherapy for brain metastases from epidermal growth factor receptor (EGFR) mutant non small cell lung cancer (NSCLC)

**DOI:** 10.18632/oncotarget.14515

**Published:** 2017-01-05

**Authors:** Qianqian Zhu, Yanan Sun, Yingying Cui, Ke Ye, Chengliang Yang, Daoke Yang, Jie Ma, Xiao Liu, Jinming Yu, Hong Ge

**Affiliations:** ^1^ Department of Radiation Oncology, The Affiliated Cancer Hospital of Zhengzhou University, Zhengzhou 450000, Henan Province, China; ^2^ Department of Radiation Oncology, The First Affiliated Hospital of Zhengzhou University, Zhengzhou 450000, Henan Province, China; ^3^ Department of Radiation Oncology, Shandong Cancer Hospital affiliated to Shandong University, Jinan, 250117, Shandong Province, China

**Keywords:** non-small cell lung cancer, brain metastases, epidermal growth factor receptor, tyrosine kinase inhibitors, radiotherapy

## Abstract

This study compared treatment outcomes between TKI monotherapy and TKI administration combined with brain radiotherapy (TKI + RT) in 133 non-small cell lung cancer (NSCLC) patients with brain metastasis (BM). We also evaluated the association of different epidermal growth factor receptor (EGFR) mutation subtypes with treatment outcome. To screen for potential variables affecting cranial progression free survival (PFS) and overall survival (OS), we performed univariate and multivariate analysis based on Cox proportional-hazards models. Median cranial PFS and OS were longer for the TKI + RT group (*n* = 67) than TKI alone group (*n* = 66). Intracranial metastasis correlated with a better median OS than extracranial metastasis. For patients with exon 21 mutations, TKI + RT yielded a better median OS and cranial PFS than TKI alone. However, there were no significant differences in median OS and cranial PFS between the two treatment groups for patients with exon 19 deletions. Thus EGFR-mutant NSCLC patients with BM could benefit more from TKI + RT than from TKI monotherapy, especially when they suffer from exon 21 mutations. However, TKI + RT confers no advantage over TKI treatment alone for patients with exon 19 deletions. These results underscore the urgent need to develop individualized disease management strategies in clinical practice.

## INTRODUCTION

Lung cancer is the major cause of cancer-related morbidity and mortality worldwide [1, 2]. Non-small cell lung cancer (NSCLC) accounts for about 85% of all lung cancer cases [3]. Of these, a prevalent subgroup is adenocarcinoma, whose incidence has been increasing in recent years [3]. Between 20–40% of NSCLC patients will develop brain metastases (BM) [4, 5]. The discovery of epidermal growth factor receptor (EGFR) mutations is one of the most promising advances for the treatment of NSCLC [3]. In Asians, ~50% of lung adenocarcinomas harbor EGFR mutations [6]. Patients with EGFR-mutant tumors are more likely to experience BM than patients with wild-type tumors [7]. Generally, patients with BM from NSCLC have poor prognosis with a median overall survival (OS) of less than 3 months for those untreated [8, 9].

Patients with BM have been historically treated with whole brain radiotherapy (WBRT) alone or in combination with surgery or stereotactic radiosurgery (SRS) [5]. Recent studies have suggested that gene profiling can help to decide the best treatment for lung cancer patients [4]. Instead of cytotoxic chemotherapy, EGFR tyrosine kinase inhibitors (TKIs) have become the first-line therapy for patients with EGFR-mutant advanced NSCLC [4, 10]. More importantly, EGFR-TKIs were also shown to be effective in treating EGFR-mutant NSCLC patients with BM, even without the upfront use of radiotherapy (RT) [11], with the median overall survival ranging from 21.9 to 26 months [2, 11]. However, development of resistance to TKIs is almost inevitable and the concentration of TKIs in cerebrospinal fluid (CSF) is lower than in plasma due to the blood brain barrier (BBB) [5, 12]. A few clinical trials indicated that EGFR-TKIs administered concomitantly with WBRT could produce a favorable overall response rate (ORR) and improve survival for patients with BM from NSCLC, especially for those who harbor EGFR mutations [13]. Additional SRS could also yield a longer OS compared with administration of TKIs alone for EGFR-mutant lung adenocarcinoma patients with BM [2].

There is no consensus on the management of patients with EGFR mutation and BM. Furthermore, the efficacy of TKI administration alone versus TKI + RT remains unclear among patients with different mutation patterns. Therefore, we performed a retrospective review to investigate whether there were differential treatment outcomes between TKI administration alone and TKI administration combined with RT in EGFR-mutant NSCLC patients with BM. We also evaluated the association of different EGFR subtypes with treatment outcome, especially exon 19 and 21 EGFR mutation patterns.

## RESULTS

A total of 215 patients were identified with 44 being excluded because of upfront use of TKI before diagnosis of BM, two due to failing to complete radiotherapy, and 19 (TKI group) due to salvage brain radiation. Furthermore, four patients with exon 18, exon 20 or compound mutations were also excluded, and 13 patients were censored. Of the remaining 133 patients, 67 were treated with TKI + RT (63 received WBRT while four received SRS) and 66 received TKI alone. The median follow-up time was 18 months (range, 4–51 months) and the median age was 56 years old (range, 28–79 years old). Overall, 79% of patients had BM at diagnosis. 58% patients in the TKI + RT group underwent salvage chemotherapy when the disease progressed. 51% patients in the TKI group received salvage chemotherapy when the disease progressed. The demographic and clinical characteristics of the patients are listed in Table 1. Baseline characteristics were well balanced in the two groups.

**Table 1:** Baseline characteristics of patients

	TKI + RT(*n* = 67)	TKI (*n* = 66)	*P*-value
EGFR Mutation Type, *n* (%)			0.336
exon 21 substitution	33 (49.3)	38 (57.6)	
exon 19 deletion	34 (50.7)	28 (42.4)	
Gender			0.800
male	30 (44.8)	31 (47.0)	
female	37 (55.2)	35 (53.0)	
TKI pattens			0.091
gefitinib	43 (64.2)	44 (66.7)	
erlotinib	24 (35.8)	22 (33.3)	
KPS			0.773
< 70	6 (9.0)	5 (7.6)	
≥ 70	61 (91.0)	61 (92.4)	
Extracranial Metastases			0.950
no	23 (34.3)	23 (34.8)	
yes	44 (65.7)	43 (65.2)	
NO. Of Brain Metastases			0.319
1 to 3	18 (26.9)	23 (34.8)	
> 3	49 (73.1)	43 (65.2)	
Age			0.187
≤ 65	56 (83.6)	49 (74.2)	
> 65	11 (16.4)	17 (25.8)	

Abbreviations: TKI + RT, tyrosine kinase inhibitors combined with radiation therapy; KPS, karnofsky performance score.

UVA of the TKI + RT group yielded a longer cranial PFS than for the TKI group, with median cranial PFS of 16.0 months and 11.5 months, respectively. In addition, the treatment of TKI + RT, KPS (≥ 70) and intracranial metastasis alone was associated with a longer OS (Table 2). MVA indicated that treatment with TKI + RT (*P* = 0.012, HR=1.888[1.150,3.100]) and intracranial metastasis alone (*P* = 0.037, HR = 1.807[1.038,3.148]) were both significant. However, KPS did not correlate with OS (*P* = 0.094).

**Table 2:** Results of univariate COX analysis of LPFS and OS for EGFR-mutant lung adenocarcinama patients with BM

	patient NO. (%)	Median PFS (mo)	*P*-value	MedianOS (mo)	*P*-value
group					
TKI + RT	67 (50)	16		22	
TKI	66 (50)	11.5	0.017	15	0.015
EGFR Mutation Type					
exon 21 mutation	71 (53)	12		16	
exon 19 deletion	62 (47)	16	0.239	19	0.324
Gender					
male	61 (46)	15		18	
female	72 (54)	14	0.913	18.5	0.687
KPS					
< 70	11 (8)	11		12	
≥ 70	122 (92)	14.5	0.451	18.5	0.032
Extracranial Metastases					
no	46 (35)	15		19	
yes	87 (65)	12	0.558	18	0.046
NO. Of Brain Metastases					
1 to 3	41 (31)	15		19	
> 3	92 (69)	12	0.286	18	0.454
Age					
≤ 65	105 (79)	14		18	
> 65	28 (21)	13	0.492	17	0.72

Abbreviations: TKI + RT, tyrosine kinase inhibitors combined with radiation therapy; KPS, karnofsky performance score; LPFS, local progression free survival; OS, overall survival; NSCLC, Non Small Cell Lung Cancer.

**Figure 1:**
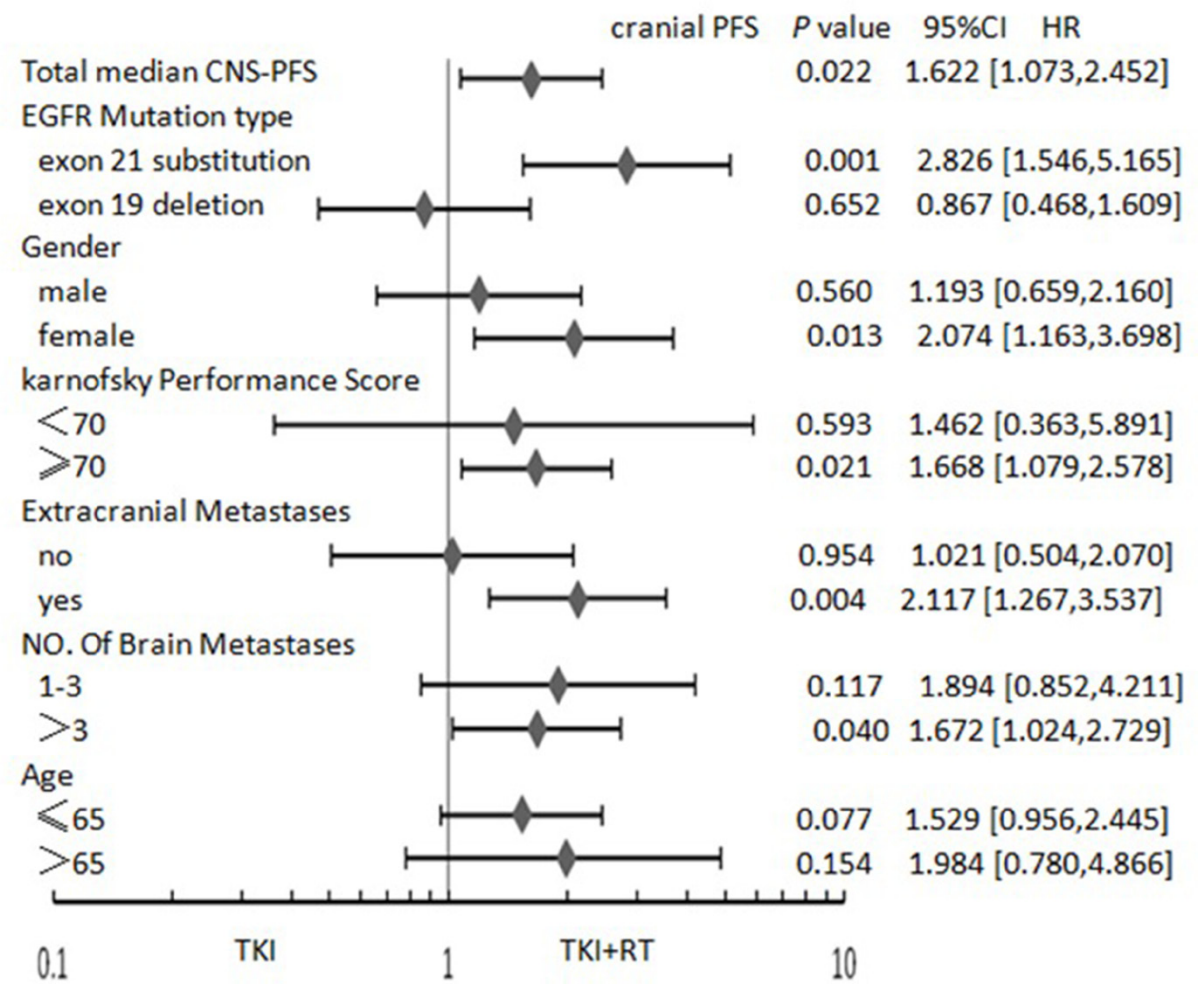
Forest plot showing hazard ratios (HR) for cranial progression-free survival (cranial PFS) and 95% confidence interval (CI) for 133 EGFR-mutant NSCLC patients with BM Abbreviations: TKI + RT, tyrosine kinase inhibitors combined with radiation therapy; KPS, Karnofsky performance score; EGFR, Epidermal Growth Factor Receptor; TKI, Tyrosine Kinase Inhibitors; NSCLC, Non Small Cell Lung Cancer.

**Figure 2:**
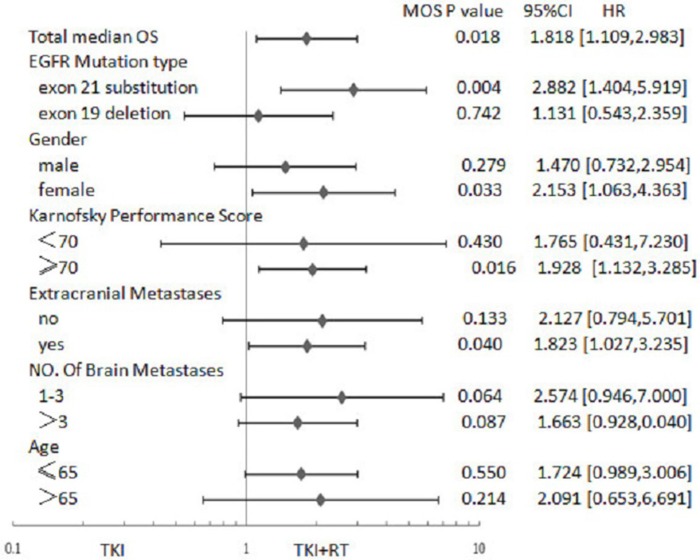
Forest plot showing hazard ratios (HR) for overall survival (OS) and 95% confidence interval (CI) for 133 EGFR-mutant NSCLC patients with BM Abbreviations: TKI + RT, tyrosine kinase inhibitors combined with radiation therapy; KPS, Karnofsky performance score; EGFR, Epidermal Growth Factor Receptor; TKI, Tyrosine Kinase Inhibitors; NSCLC, Non Small Cell Lung Cancer.

**Figure 3:**
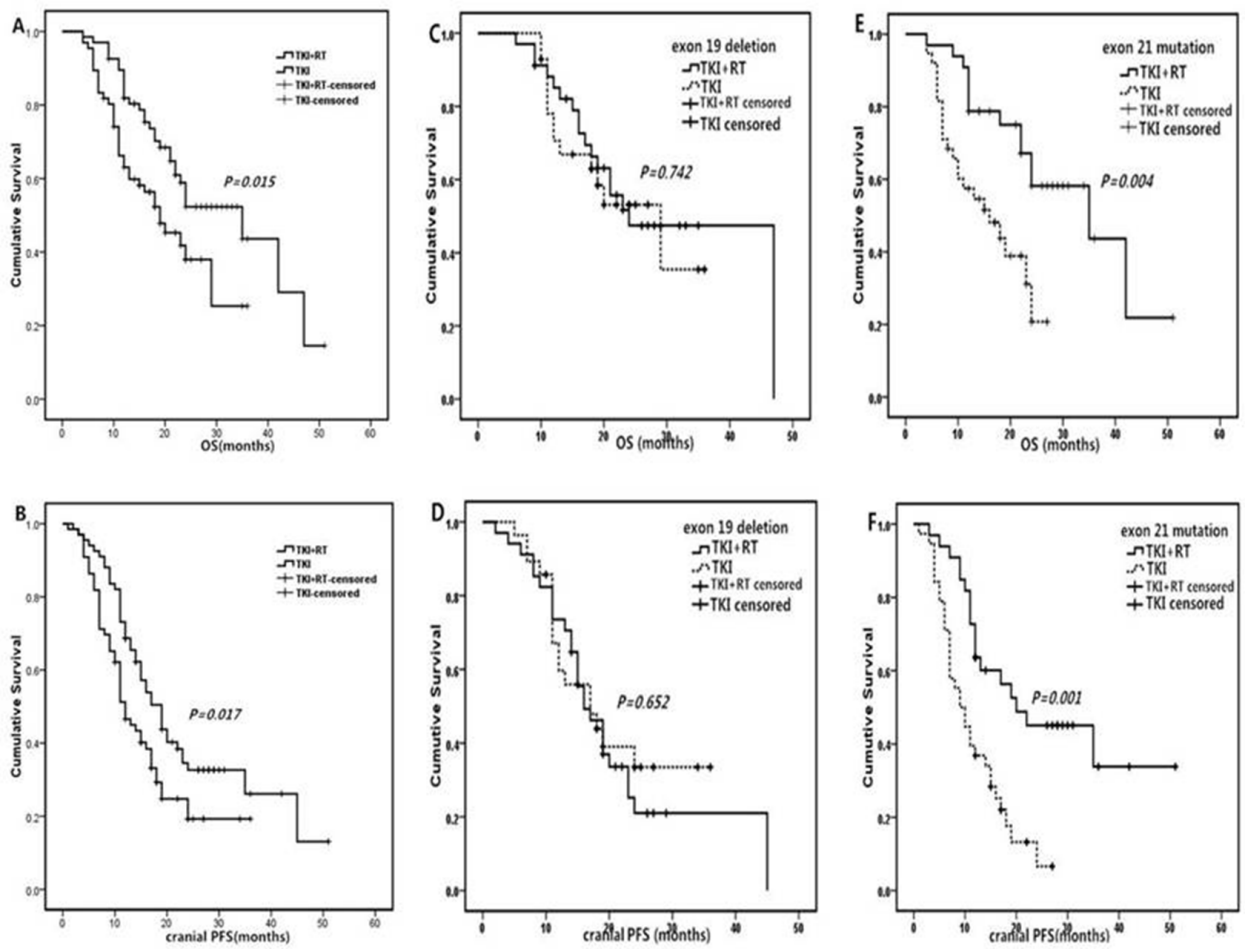
Kaplan-Meier curves of cranial PFS and OS between TKI + RT and TKI alone (**A**) OS in enrolled 133 patients; (**B**) cranial PFS in enrolled 133 patients; (**C**) OS in 19 deletion subgroup; (**D**) cranial PFS in 19 deletion subgroups; (**E**) OS in 21mutation subgroup; (**F**) cranial PFS in 19 deletion subgroups Abbreviations: PFS, progression free survival; OS, overall survival.

As shown in Figure 1, for patients with exon 21 mutations, female, KPS (≥ 70), number of brain metastases (> 3), and extracranial metastasis, the TKI + RT group faired better than the TKI group in terms of cranial PFS. In the case of patients with exon 21 mutation, female, KPS (≥ 70) and extracranial metastasis, median OS in TKI + RT group was longer than those treated with TKI alone (Figure 2). When EGFR mutations occurred in exon 21, the TKI group again showed inferior results than the TKI + RT group, with a shorter cranial PFS (9.5 vs 14 months, *P* = 0.001) and OS (13.5 vs 22 months, *P* = 0.004). For patients with exon 19 deletions, there were no significant differences between the two groups in terms of cranial PFS (16.0 vs 16.0 months, *P* = 0.652) and OS (18.5 vs 20.5 months, *P* = 0.742) (Figure 3).

## DISCUSSION

With the rapid integration of molecular profiling into clinical practice, EGFR mutation positive NSCLC has been typified by having high and durable response to TKI [11]. Previous reports have shown that the median cranial PFS ranged from 11.2 to 26 months and median OS ranged from 11.5 to 35 months for EGFR-mutant patients with BM from NSCLC [2, 13, 15]. EGFR-mutant patients show partial response or achieve relative stability after TKI treatment but are not cured; therefore, additional treatment approaches could improve the disease management strategies for this cohort.

In our study, median cranial PFS (16.0 vs 11.5 months, *P* = 0.017) and median OS (22 vs 15 months, *P* = 0.015) were longer for the TKI + RT group than for the TKI group, and KPS (≥ 70) and intracranial metastasis were only associated with superior OS when measured by UVA. Upon MVA, the KPS was not significant; however, treatment method (*P* = 0.012) and extracranial metastasis (*P* = 0.037) remained significant.

Previous studies reported, similar findings [9, 16, 17]. For example, Zeng YD *et al*. conducted a retrospective study to compare the efficacy of TKI administration plus concomitant WBRT against TKI administration alone. They analyzed data from 90 patients with BM from NSCLC and found that TKI treatment alone was associated with poorer prognosis than TKI treatment plus RT, with a shorter median cranial PFS (4.17 vs 7.12 months, *P* = 0.001) and OS (14.83 vs 23.4 months, *P* = 0.002) [9]. A meta analysis reported that the objective response rate (ORR), PFS and two-year OS of intracranial disease were improved by treatments with upfront cranial radiotherapy [16]. Previous studies included patients of unknown EGFR mutation status. On the other hand, all the patients enrolled in our study were EGFR positive. That might explain the better cranial PFS we observed. In addition, another study compared the survival outcome of upfront TKI vs upfront RT, finding an inferior OS for upfront TKI than for upfront RT (*P* = 0.01) [17]. Yet, other studies arrived at different conclusions [2, 18]. For example, Jiang T *et al*. reported that OS was a shorter when patients were treated with WBRT combined with TKI compared with TKI alone (*P* = 0.049) and found no significant difference in cranial PFS between the two groups (*P* = 0.232) [18]. This study was also a retrospective research, so the consequence of bias was inevitable. Besides, intracranial metastasis only had a superior OS (19 vs 18 months, *P* = 0.037) than extracranial metastasis in our study. Lasty, Gerdan *et al*. showed that when one extracranial organ is involved (or none), survival is improved compared to when two or more organs are involved [19].

EGFR mutations are usually oncogene-driven, occurring within exons 18–21. Exon 19 deletions and exon 21 mutations (L858R) account for about 90% of these mutations [20, 21]. Previous studies showed different ORR between the two subtypes for patients with BM receiving TKI alone [11, 21, 22]. A phase 2 trial revealed a longer PFS (*P* = 0.003) and OS (*P* = 0.025) in lung adenocarcinoma patients harboring exon 19 deletions with BM than in patients harboring exon 21 mutations [11]. Interestingly, NSCLC patients with exon 19 deletions have more and smaller brain lesions with smaller brain edema than patients with wild-type EGFR. No significant difference was found between patients with exon 21 point mutations and those with wild-type EGFR [23]. On the other hand, we performed a subgroup analysis in our current study and found that OS (22 vs 13.5 months, *P* = 0.004) and cranial PFS (14 vs 9.5 months, *P* = 0.001) were longer for the TKI + RT group than for the TKI alone group for lung adenocarcinoma patients with BM harboring exon 21 mutations. However, there were no significant differences in OS (20.5 vs 18.5 months, *P* = 0.742) and cranial PFS (16.0 vs 16.0 months, *P* = 0.652) between the two groups for lung adenocarcinoma patients with BM harboring exon 19 mutations. Therefore, TKI administration alone may be inadequate to treat patients with exon 21 mutations and combination therapies may provide additional benefits.

Several mechanisms have been proposed to explain the combinatorial effect of TKI + WBRT on EGFR-mutant NSCLC patients with BM [24–27]. For example, erlotinib may decrease the duration of cell cycle arrest, block anti-apoptosis pathways, and accelerate cellular repopulation and reduce DNA damage repair [14, 25]. On the other hand, radiotherapy can enhance the permeability in blood brain barrier (BBB), which increases the effective concentration of TKI [26]. Finally, radiation reduces the effects of the T790M mutation, which is known to confer resistance [27, 28]. Although it was reported that TKI combined with brain radiation did not increase the incidence of adverse events [13, 29, 30], some studies suggested such treatment might cause more neurological adverse events compared to TKI alone in the long term [16].

At present, the problem of treatment with TKI with or without RT for patients with BM from NSCLC remains controversial. Owing to decreasing neurotoxicity, SRS is often an optimal option for patients with low tumor volume and limited number of BM [24]. However, for asymptomatic patients with a limited number of small brain lesions, upfront TKI without RT is always the preferred treatment, even though it is less likely to stop disease progression in the absence of RT [2, 24]. According to a previous study, it is reasonable to continue EGFR TKI beyond progressive disease (PD) in patients with minor disease progression, particularly when they are asymptomatic [24]. Unfortunately, a benefit of only 3.1 months in increased OS was obtained by further use of TKI treatment [31]. Usually, PD is defined according to RECIST criterion based on radiological diagnosis. However, approximately 50% of patients harbor the T790M mutation in addition to EGFR-mutant resistance [28]. Indeed, T790M mutations were found in plasma up to four months earlier than PD [24]. Indeed, the definition of EGFR-mutant resistance remains unclear. Detection of blood biomarkers and radiological examination are encouraged to screen patients with TKI resistance in a timely manner.

The present study is limited by its retrospective nature. Due to lack of randomization and the small number of patients treated with SRS (four), bias might artificially yield a prolonged OS for the TKI + RT group [2, 32]. Moreover, adverse effects were not investigated due to the lack of clinical data. Furthermore, the difference between two types of TKI treatment was not analyzed. However, it was previously demonstrated that there are no differences in PFS and OS between using erotinib and gefitinib to treat patients with lung adenocarcinoma [33].

EGFR-mutant abundance has been recognized as a prognosis factor in lung cancer. Patients with high abundance of EGFR mutations receiving TKI treatment had superior ORR and OS than those with wild-type EGFR and low EGFR-mutant abundance [34]. We measured the abundance of EGFR mutations in only 31 of the patients. However, to avoid statistical bias, this factor was not analyzed in our study.

In conclusion, EGFR-mutant NSCLC patients with BM could benefit more from TKI combined with brain radiation than from TKI monotherapy. Patients with exon 21 mutations can also benefit more from TKI + RT rather than TKI alone. However, no benefit was measured from TKI + RT for patients with exon 19 deletions. Because complete response is difficult to achieve by TKI alone treatment, new therapies and combinations should be introduced. With approval of 3rd generation inhibitors, management paradigm will be faced with a great change. Multiplexed clinical testing, such as measuring dynamic changes in plasma, EGFR mutation status, as well as performing molecular imaging, might allow developing individualized management strategies in clinical practice.

## MATERIALS AND METHODS

### Patients

From January 2011 to April 2015, 215 EGFR-mutant lung adenocarcinoma patients with BM were identified at the Affiliated Cancer Hospital of Zhengzhou University and The First Affiliated Hospital of Zhengzhou University. This study was approved by our institutional review board and written informed consent was obtained from each patients.

EGFR mutations were detected by direct sequencing of DNA extracted from the paraffin-embedded tumor samples. The eligibility criteria for this study were: (1) EGFR-mutant lung adenocarcinoma with BM, and (2) treatment with either TKI (erlotinib or gefitinib) alone or TKI + RT. On the other hand, the exclusion criteria were: (1) upfront use of EGFR-TKI before BM was diagnosed; (2) failing to finish the plan for WBRT; (3) exon 18, exon 20 or compound mutations; (4) TKI administration + salvage RT.

### Treatments

Patients were treated with TKI including erlotinib (150 mg/ day) or gefitinib (250 mg/day) from diagnosis of BM until TKI resistance or unacceptable toxicity reaction to TKI administration was developed. The TKI + RT group received either WBRT or stereotactic radiotherapy (SRS). For WBRT, the total dose varied between 30 and 40 Gy in 10 to 20 fractions, 5 days per week. Radiation was delivered using a linear 6 MV photon accelerator. The median marginal dose of SRS was 20 Gy (range, 15–24 Gy). The dose and duration of TKI treatment were the same for both groups with and without RT. According to response evaluation criteria in solid tumors (RECIST) 1.1 [14], tumor response was assessed by magnetic resonance imaging (MRI).

### Statistical analysis

Chi-squared test was used to compare the baseline characteristics of patients between the two groups. In our study, baseline variables included age at diagnosis of BM, gender, TKI treatment types, Karnofsky performance score (KPS), EGFR mutation pattern, number of brain lesion, and extracranial metastasis. The primary end point was overall survival (OS), and the secondary end point was cranial progression-free survival (cranial PFS). Cranial PFS of intracranial lesions was measured from the date of the diagnosis of BM until intracranial progression. If brain metastasis progressed before the date of last follow-up or death from any cause, cranial PFS was calculated from the time of BM determination until death from any cause or the last follow-up. OS was measured from the date of the diagnosis of BM until death. If brain metastasis progressed before the date of last follow-up or death from any cause, OS was calculated from the time of BM determination until death from any cause or the last follow-up. Data of death were collected from medical records or through telephone interviews with relatives. The Kaplan–Meier method was used to generate survival curves. The log-rank test was used to measure differences between the two groups. To screen potential variables affecting cranial PFS and OS, univariate analyses (UVA) and multivariate analyses (MVA) based on Cox proportional-hazards models were performed. Two-sided *P*-values less than 0.05 were considered statistically significant. Statistical analyses were performed using SPSS software version 20.0.
